# Economic Evaluation in Neurological Physiotherapy: A Systematic Review

**DOI:** 10.3390/brainsci11020265

**Published:** 2021-02-19

**Authors:** David García-Álvarez, Núria Sempere-Rubio, Raquel Faubel

**Affiliations:** 1 Physiotherapist, Faculty of Physiotherapy, Universitat de València, 46010 Valencia, Spain; dagaral4@alumni.uv.es; 2 Department of Physiotherapy, Universitat de València, 46010 Valencia, Spain; raquel.faubel@uv.es; 3 Clinical Biomechanics Research Unit (UBIC), Department of Physiotherapy, Universitat de València, 46010 València, Spain; 4 Joint Research Unit in Biomedical Engineering, IIS La Fe-Universitat Politècnica de València, 46026 Valencia, Spain; 5 PTinMOTION, Physiotherapy in Motion, Multispeciality Research Group, Department of Physiotherapy, Universitat de València, 46010 Valencia, Spain

**Keywords:** cost-benefit analysis, economics, medical, physical therapy modalities, neurology

## Abstract

This systematic review was carried out to compile and assess original studies that included economic evaluations of neurological physiotherapy interventions. A thorough search of PubMED, Cochrane and Embase was developed using keywords such as health economics, neurological physiotherapy and cost analysis, and studies published during the last six-year term were selected. A total of 3124 studies were analyzed, and 43 were eligible for inclusion. Among the studies analyzed, 48.8% were interventions for stroke patients, and 13.9% were focused on Parkinson’s disease. In terms of the countries involved, 46.5% of the studies included were developed in the UK, and 13.9% were from the USA. The economic analysis most frequently used was cost-utility, implemented in 22 of the studies. A cost-effectiveness analysis was also developed in nine of those studies. The distribution of studies including an economic evaluation in this discipline showed a clear geographic dominance in terms of the pathology. A clear upward trend was noted in the economic evaluation of interventions developed in neurological physiotherapy. However, these studies should be promoted for their use in evidence-based clinical practice and decision-making.

## 1. Introduction

Evidence-based medicine, defined by David Sacket and Gordon Guyatt as a process whose objective is to obtain and apply the best scientific evidence in the exercise of routine medical practice [1], requires the conscientious, judicious and explicit use of the best available evidence in decision-making regarding the health care of patients [2]. This is a concept equally applicable in other health areas; thus, so-called evidence-based health care has emerged. Physiotherapy, therefore, also needs to be implemented through the use of evidence-based medicine. Finch et al. [3], in their guidelines for decision-making in clinical practice, report that the three most relevant paradigms are the WHO International Classification of Functioning, Disability and Health (ICF), health-related quality of life (HRQL) and the costs of the interventions evaluated. The two latter paradigms, however, are closely related, since HRQL is used, together with well-being and health status, in cost-utility analyses of interventions. Accordingly, we found that health economics was referenced in two of the three paradigms considered fundamental in the evidence-based practice of physiotherapy [4].

Physiotherapy is concerned with human function and movement and maximizing physical potential. It includes services provided to individuals and populations to develop, maintain and restore maximum movement and functional ability throughout the lifespan, and it is concerned with identifying and maximizing quality of life and movement potential within the spheres of promotion, prevention, treatment/intervention and rehabilitation. It uses physical means to promote, maintain and restore a biopsychosocial model of the individual’s health. It relies on scientific evidence to discuss, evaluate and review its practices. [5]. However, despite the need for economic evaluations in evidence-based clinical practice, there are still a reduced number of scientific publications on physiotherapy in Spain that include economic analyses, and their methodological quality is poor [6].

Economic evaluations of health interventions involve a comparative analysis of numerous interventions relating the differences in costs to the differences in the effects of such interventions. These economic assessments are mainly implemented using four methods: cost-minimization, cost-benefit, cost-effectiveness and cost-utility. The cost-minimization analysis assumes that interventions achieve equivalent benefits and seeks to establish which intervention is associated with lower resource consumption. Cost-benefit analyses express both the costs and the effects of interventions in monetary terms, making it easier to compare them with the costs of such interventions. On the other hand, the cost-effectiveness analysis estimates the incremental cost per unit of effects when considering effects in common with the interventions being compared, and it is based on natural units. Ultimately, the cost-utility analysis is considered by some authors to be a variant of the cost-effectiveness analysis, in which the unit of effect is a generic measure of health, such as quality-adjusted life-years (QALY), that takes into account both the health-related quality of life and the increase in life expectancy obtained as a result of the intervention [7].

One of the issues to consider in an economic evaluation of interventions is the perspective of the study, which is the point of view adopted when deciding which types of costs and health benefits will be included in the economic evaluation. Typical viewpoints are those of the patient, hospital/clinic, healthcare system or society. Therefore, a health economic evaluation can be conducted from one or more perspectives, such as the societal perspective, the healthcare payer perspective or the patient perspective [8]. The perspective used in a study is determined by the purposes of the research and also by methodological issues. Likewise, the perspective determines which types of costs are included in the analysis because they are relevant to their interests. For instance, societal perspectives and patient perspectives include indirect costs (such as time lost during transportation to the health center) and intangible costs (such as pain and suffering) besides other types of costs included in payers’ perspective, such as direct medical costs (hospitalization, diagnostic procedures, outpatient visits, etc.). Subsequently, the perspective taken in an economic analysis can have an important influence on how an intervention is assessed and the results obtained and interpreted [9,10].

Neurological disorders and conditions affect the functioning of an individual and produce disabilities or limit activities and social participation. Neurological disorders, neurological impairments and sequelae constitute over 6% of the global burden of disease. In addition to causing mortality and disability, people with neurological diseases experience individual suffering, suffering in their families and their community and social and economic losses. This results in a decrease in their productivity and quality of life. According to a European study that partially included direct non-medical costs and indirect costs and omitted intangible costs, the annual economic cost of neurological diseases amounted to €139 billion in 2004 [11].

The field of neurology in physiotherapy gathers the largest volume of controlled studies published, and it represents 15.4% of the total evidence; it is only outscored by those related to traumatology and orthopedics, representing 33.3% [6]. Even so, there are no studies that have linked the current situation of neurology in physiotherapy with economic studies. The objective of this systematic review is to collect and evaluate from the existing literature the evolution, magnitude and characteristics of studies that have included economic evaluations of interventions in the field of neurological physiotherapy.

## 2. Materials and Methods

This study follows the guidelines of the Preferred Reporting Items for Systematic Reviews and Meta-Analyses (PRISMA) [12].

### 2.1. Bibliographic Search

A systematic review was executed using a structured electronic search following the procedure proposed by PRISMA in PubMED, Cochrane and Embase databases. The search used keywords such as health economics, neurological physiotherapy and cost analysis and related words such as neurophysiotherapy, neurorehabilitation, neurological rehabilitation, physical rehabilitation, cost-effectiveness, cost effectiveness, cost-utility, cost utility, cost-benefits and cost benefits. A manual search was also effectuated, including the references of the articles found and related articles.

### 2.2. Selection Criteria

The articles included were studies published between January 2014 and December 2019 (both inclusive) comprising economic evaluations of at least one neurological physiotherapy intervention published in any language. Studies excluded were those conducted in animal models, those which analyzed non-neurological pathologies derived from others that were neurological (e.g., pressure ulcers), those performed in subjects with intellectual disabilities, those performed in heterogeneous groups that included subjects without neurological pathologies and studies published without results (e.g., protocols). All of the identified articles were independently analyzed by at least two researchers from the present study, and the final selection of the articles to be included was made by consensus.

### 2.3. Data Extraction

We used a data extraction form to collect data on model type. Information was extracted from each study related to the year of publication, country in which it was performed, target pathology of the intervention, type of analysis implemented and the perspective from which the economic evaluation was implemented. The identified studies were analyzed, and data were extracted independently by two study authors.

## 3. Results

### 3.1. Search Results

**Figure 1:**
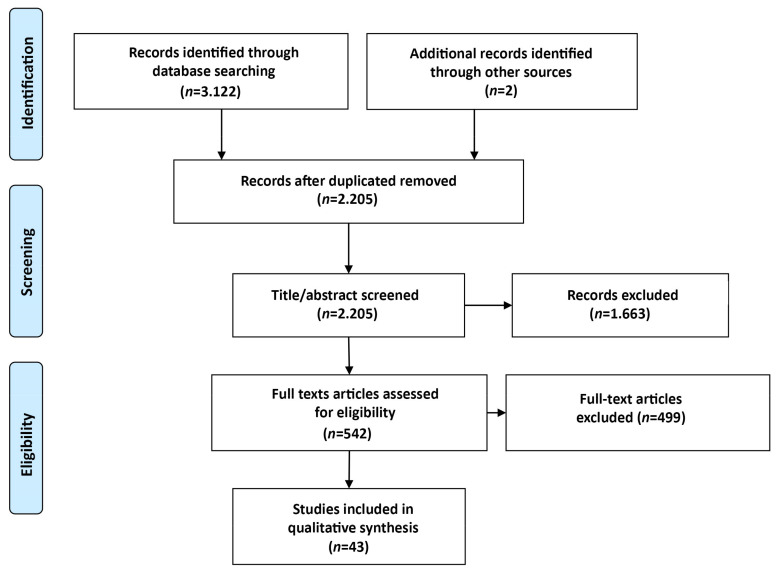
Flowchart of study selection process.

A total of 3124 articles were identified in the structured search of the three databases and the manual search, which were then analyzed and screened (Figure 1). After discarding duplicates, 2205 studies were evaluated by reading the title, abstract and keywords, and 1663 studies were rejected. Subsequently, 542 articles were full-text analyzed, of which 499 were discarded because they failed to meet the selection criteria. Ultimately, 43 articles were included in this review.

### 3.2. Description of Studies Included

The characteristics of the studies included are detailed in Table 1. The pathologies covered by the largest number of studies were stroke with 21 studies (representing 48.8%), Parkinson’s disease with 6 studies (13.9%) and multiple sclerosis with 5 studies (11.6%). Only one study was published on the other eight pathologies, with the exception of complex regional pain syndrome with two studies [13,14]. On the other hand, two articles were found that included heterogeneous groups of patients with neurological disorders [15,16]. 

**Figure 2:**
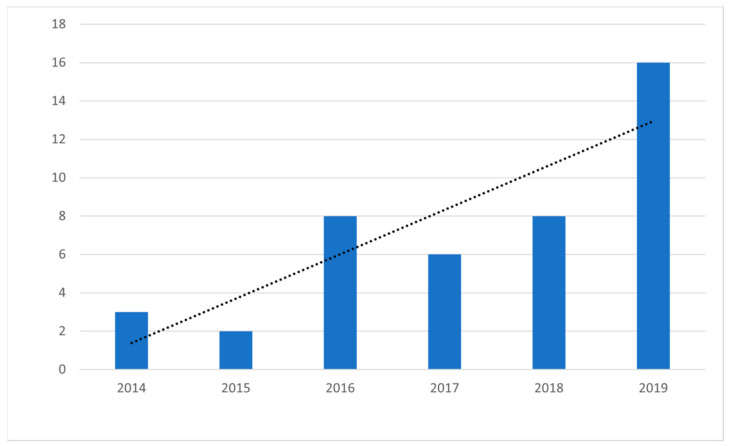
Year in which studies were conducted.

An upward trend was noted in the 43 articles included in this review that were published between 2014 and 2019, as shown by the trend line in Figure 2. The number of studies varied across this period, with a minimum of two studies in 2015, progressively increasing to 16 conducted in 2019, with a slight downturn in 2017. In order to compare the magnitude of economic evaluations performed in neurological physiotherapy, two different periods were created during the time of the study. The number of studies more than doubled over the two periods: 13 studies were published in the first three-year period studied (2014–2016), compared to 30 studies published in the second triennium (2017–2019).

**Figure 3:**
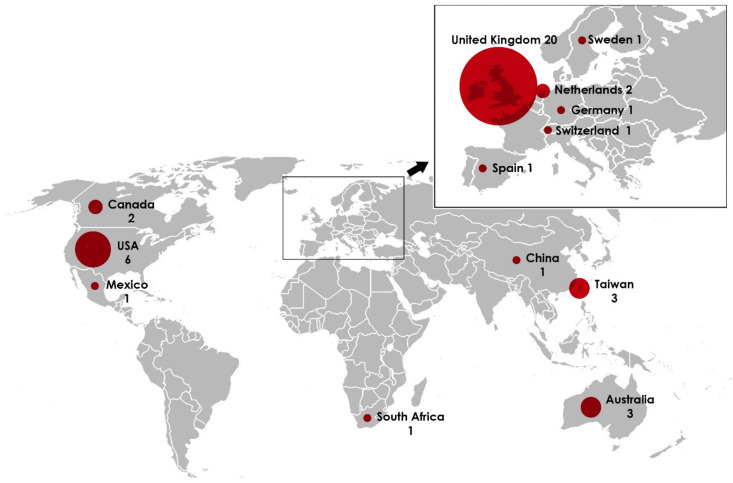
Geographical distribution of the studies included.

These interventions were executed in 13 different countries (Figure 3) and showed the geographical dominance of two countries, namely the United Kingdom and the US, with 20 studies and 6 studies respectively. The remaining papers were distributed among the remaining 11 countries, with between one and three articles published per country. Thus, 46.5% of the studies included in the review were conducted in the United Kingdom and 13.9% in the United States.

Regarding the type of economic analysis carried out, 30 studies had effectuated comprehensive economic evaluations. The cost-utility analysis was the most frequently used type of analysis, appearing in more than 70% of these studies, either alone or in combination with a cost-effectiveness analysis. The cost-effectiveness analysis was developed in half of the studies conducting comprehensive evaluations. Only one study performed a cost-minimization analysis [47], and another study conducted a cost-benefit analysis [45]. On the other hand, 13 studies effectuated partial economic evaluations such as a cost-consequence analysis (in 11 studies) or cost description [16,44].

**Figure 4:**
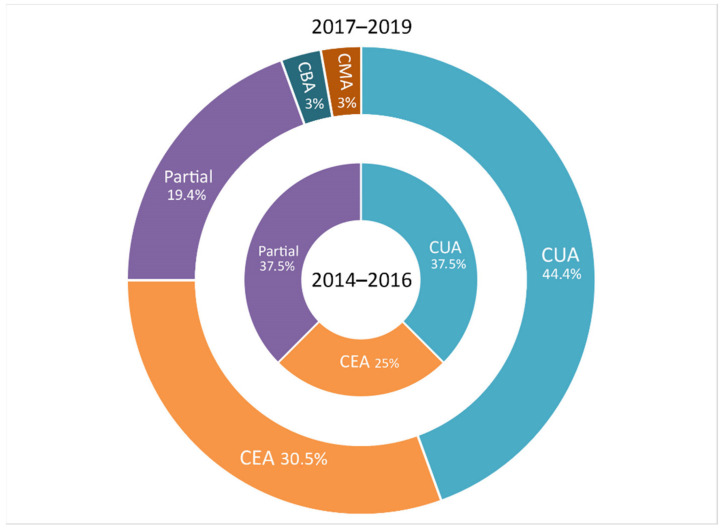
Type of economic evaluations carried out in the studies included according to the triennium. Note: CUA: cost-utility analysis; CEA: cost-effectiveness analysis; CBA: cost-benefit analysis; CMA: cost minimization analysis.

The number of published studies with various economic analyses (Figure 4) exhibited a clearly upward progression for studies that included a cost-effectiveness analysis. It went from four studies in the first triennium, 2014–2016 (25% of the published studies), to 11 studies in the second three-year period, 2017–2019 (30.5%). An upward trend was also found for the studies that included a cost-utility analysis, going from six studies in the first triennium (37.5% of the published studies) to 16 in the second period (44.4%). In contrast to this, the percentage of partial evaluations dropped significantly between the first and second trienniums, being reduced from 46% of studies in the first to only 23.3% in the second.

If we focus on the relationship between country and pathology, the five studies on multiple sclerosis were executed in the United Kingdom. Similarly, the two studies on complex regional pain syndrome were conducted in the Netherlands. On the other hand, when considering stroke, the most studied disease, it should be noted that of the 22 studies that analyzed neurological physiotherapy interventions in connection with this disease, 8 were performed in the United Kingdom and 3 in Taiwan.

Focusing on the relationship between economic analyses and countries in which the studies were carried out, all of the comprehensive economic evaluations conducted in the UK included cost-utility studies, while only one of six US studies did.

Effects of the intervention analyzed in each study were measured through different outcome variables according to the type of analysis, type of intervention, pathology of the participants and the objectives of the study. Consequently, QALY was measured in all the studies conducting cost-utility analysis [13,14,17,18,20,21,22,27,28,31,32,34,39,40,41,42,43,46,49,50,51,54]. Other health outcomes were used including HRQL [14,27,28,31], daily life activities [22,52] or pain [13]. In other studies, we could find variables related to the pathology in the study, including balance and gait speed [40] and number of falls avoided due to the intervention in studies for Parkinson participants [32,43,47]. Likewise, the effect of interventions for stroke patients was measured in some studies using specific clinical scales for stroke [38,52], i.e., the NIHSS scale (US National Institutes of Health Stroke Scale). Lastly, other types of outcome variables were directly related to the healthcare system such as readmissions [30] or hospitalizations per day [53].

In terms of the perspective of the health economics evaluation, 16 of the 43 studies included in the review did not explicitly state the perspective of the cost-analysis implemented in the study. Nevertheless, in most of the studies, the perspective was assumed for our review purposes based on the types of costs included in the analysis. As shown in Table 1, only 14% of the studies included the social perspective [14,16,17,34,40,43], while 58% included the healthcare payer perspective, and 30% were based on the healthcare payer perspective but also included other non-health costs. The patient perspective was only used in two of the studies included in the review [46,48]. Nevertheless, some types of costs related to the patient perspective had been included in five other studies using the health funder perspective: for instance, co-payments [25,37] or travel expenses paid by the patient for transportation to the health institution for outpatient visits [13,31,44].

## 4. Discussion

In the six-year period analyzed (2014–2019), 43 neurological physiotherapy studies were identified that included economic evaluations. The results show a clear upward trend in the publication of studies by year in addition to a predominance of the United Kingdom over other countries. Findings also highlight how stroke is addressed more often than other pathologies, and that the cost-utility analysis is the more frequently used. 

Although previous reviews have assessed economic evaluations in specific fields such as human papillomavirus (HPV) self-sampling programs [56], orthopedic surgery [57] and sleep medicine [58], to our knowledge, this is the first systematic review in neurological physiotherapy.

The relevance of stroke seems to be related to the study of disease burden, whereby, in 2016, the likelihood of suffering this disease was as high as 24.9% [59,60]. That year, there were 13.7 million new cases, of which one-fifth resulted in death [61], and 35–71% of survivors developed disability [60,62,63,64,65]. The same studies disclosed that there were between 79.5 and 80.1 million prevalent cases, and that it was the disease with the second-highest burden of disability-adjusted life-years (DALYs), ranking after myocardial infarction [66,67]. Importantly, it also especially affected working-age subjects [59], and in the United Kingdom alone, the annual expenditure deriving from this pathology was approximately GBP 26 billion [68].

This review reveals studies which allege having performed cost-effectiveness analyses; however, after reviewing the methodology described, these have been considered partial economic analyses. Most of them are cost-consequence analyses wherein the authors have studied the differences in costs between two therapeutic alternatives and have also analyzed the difference in effects, although without relating the two variables. Among the comprehensive economic evaluations, there is a high proportion of studies (73.3%) that have included a cost-utility analysis, which is consistent with the methodological recommendations that propose the use of cost-utility analyses [5,69] since it allows, among other advantages, to compare effects of interventions in different disciplines, with varying clinical results. However, in nine of these studies, in addition to a cost-utility analysis, a cost-effectiveness analysis was carried out. This makes it possible to analyze the association of the costs of the intervention with measurements of the effects of the intervention, such as patient-reported health outcomes (PROs), among which we find HRQL, daily life activities, or variables related to the healthcare system like readmissions avoided [30] or days in hospital avoided [53]. Measurement of the effects based on these variables as performed in cost-effectiveness analyses can make the collection of the effect information more affordable, while a more direct interpretation of the results of the economic evaluation by the decision makers is possible.

The high percentage of studies conducted in the United Kingdom could be related to the existence of a public, executive and non-departmental body such as the National Institute for Health and Care Excellence (NICE), whose principles include the economic assessment of interventions [70] and which arose as a response to the “Health and Social Care Act 2012”, which requires NICE to consider the balance between the benefits and costs of providing health or social care services in England [71].

The results of this review show a clear increase in the number of economic evaluations in the study period that could also be maintained in 2020 since, after reproducing the search strategy, 783 published articles were found in the first four months of 2020 compared to 1973 published throughout 2019. This represents an 18% increase, although the circumstances affecting scientific production in 2020 might affect this prediction.

One of the limitations of our study was that the methodological quality of the studies covered was not analyzed since the aim of the review was to analyze the magnitude of published studies. Another limitation was the variability in terminology in the types of economic analyses used by the various published studies. Lastly, the full text of 4 of the 43 studies could not be analyzed, although in these cases, the abstract contained sufficient information to be included in the review. Strengths of this systematic review included the fact that it was performed using the parameters established by PRISMA using three of the most important databases in physiotherapy.

Undoubtedly, the relevance of physiotherapy has increased in recent years, specifically in neurology, which also implies an increase in the variety of interventions. Therefore, future studies in this field are needed, which also should include economic evaluations for its application in clinical practice based on evidence and decision making.

## 5. Conclusions

Economic evaluations are a fundamental pillar in evidence-based clinical practice, involving a comparative analysis of interventions relating the differences in costs to the differences in effects of such interventions. This systematic review has identified 43 studies on neurological physiotherapy that included economic evaluations, with a clear predominance of stroke as the pathology addressed in the interventions analyzed and a large number of studies conducted in the United Kingdom. Cost-utility studies have been the most represented, either alone or together with cost-effectiveness analyses. In terms of the evolution, there has been a clear increase in the number of studies published in recent years, and this progression is expected to continue. Economic evaluations of neurological physiotherapy interventions need to be promoted urgently in order to provide higher-quality publications and enable their transfer to clinical practice and decision-making.

## Figures and Tables

**Table 1:** Characteristics of the studies included in the systematic review.

Author	Year	Country	Pathology	Type of Analysis	Perspective	Effect Measurement
Ademi et al. [17]	2016	Switzerland	Radiculopathy	Cost-utility analysis	Healthcare payer/Societal	QALY ^a^
Adie et al. [18]	2016	United Kingdom	Stroke	Cost-utility analysis	Payer	QALY
Alberts et al. [19]	2019	United States	Concussion	Partial evaluation	Healthcare payer	-
Allen et al. [20]	2019	Canada	Stroke	Cost-utility analysis	Payer	QALY
Ashburn et al. [21]	2019	United Kingdom	Parkinson	Cost-utility analysis	Payer	QALY
Barnhoorn et al. [13]	2018	Netherlands	Complex regional pain syndrome	Cost-effectiveness analysis Cost-utility analysis	Healthcare payer + travel expenses	Disability and pain QALY
Bhattarai et al. [22]	2018	United Kingdom	Stroke	Cost-effectiveness Cost-utility	Payer	NEADL ^b^ QALY
Bustamante et al. [23]	2016	Mexico	Stroke	Partial evaluation	Healthcare payer	-
Butler et al. [24]	2017	United States	Stroke	Partial evaluation	-	-
Chen et al. [25]	2019	Taiwan	Stroke	Cost-effectiveness analysis	Healthcare payer + co-payment	Years of life
Christofi et al. [26]	2016	United Kingdom	Stroke	Partial evaluation	Healthcare payer	-
Clarke et al. [27]	2016	United Kingdom	Parkinson	Cost-effectiveness analysis Cost-utility analysis	Payer	HRQL ^c^ QALY
Collins et al. [28]	2018	United Kingdom	Stroke	Cost-effectiveness analysis Cost-utility analysis	Healthcare payer	HRQL QALY
Cooney et al. [29]	2016	United Kingdom	Stroke	Partial evaluation	Healthcare payer	-
Crotty et al. [30]	2016	Australia	Stroke	Cost-effectiveness analysis	Healthcare payer	Readmission avoided
Dean et al. [31]	2018	United Kingdom	Stroke	Cost-effectiveness analysis Cost-utility analysis	Healthcare payer + travel expenses	HRQL QALY
den Hollander et al. [14]	2018	Netherlands	Complex regional pain syndrome	Cost-effectiveness analysis Cost-utility analysis	Societal	HRQL QALY
Farag et al. [32]	2015	Australia	Parkinson	Cost-effectiveness analysis Cost-utility analysis	Healthcare payer	Falls and mobility QALY
Farr et al. [33]	2019	United Kingdom	CCP ^d^	Partial evaluation	Healthcare payer	
Freeman et al. [34]	2019	United Kingdom	Multiple sclerosis	Cost-utility analysis	Payer/Societal	QALY
George et al. [35]	2019	United States	Stroke	Partial evaluation	Healthcare payer	
Hesse et al. [36]	2014	Germany	Stroke	Partial evaluation	Healthcare payer	
Hester et al. [15]	2017	United States	Heterogeneous group	Partial evaluation	Healthcare payer	-
Hind et al. [37]	2017	United Kingdom	Duchenne muscular dystrophy	Partial evaluation	Healthcare payer + co-payment	-
Ho et al. [38]	2019	Taiwan	Stroke	Cost-effectiveness analysis	Healthcare payer	Clinical stroke scales
Hunter et al. [39]	2017	United Kingdom	Stroke	Cost-utility analysis	Payer	QALY
Joseph et al. [40]	2019	Sweden	Parkinson	Cost-effectiveness analysis Cost-utility analysis	Societal	Balance and gait QALY
Juckes et al. [41]	2019	United Kingdom	Multiple sclerosis	Cost-utility analysis	Healthcare payer	QALY
Lamb et al. [42]	2018	United Kingdom	Dementia	Cost-utility analysis	Payer	QALY
Li et al. [43]	2015	United States	Parkinson	Cost-effectiveness analysis Cost-utility analysis	Societal	Fall avoided QALY
Llorens et al. [44]	2014	Spain	Stroke	Partial evaluation	Healthcare payer + travel expenses	-
Louw et al. [45]	2019	South Africa	Stroke	Cost-benefit analysis	Payer	-
McClrurg et al. [46]	2018	United Kingdom	Multiple sclerosis	Cost-utility analysis	Healthcare payer/Patient	QALY
Morris et al. [47]	2017	Australia	Parkinson	Cost-minimization analysis	Healthcare payer	Fall avoided
Paganoni et al. [48]	2019	United States	ALS ^e^	Cost-effectiveness analysis	Healthcare payer/Patient	Perceived utility
Renfrew et al. [49]	2019	United Kingdom	Multiple sclerosis	Cost-utility analysis	Payer	QALY
Rodgers et al. [50]	2019	United Kingdom	Stroke	Cost-utility analysis	Payer	QALY
Rodgers et al. [51]	2019	United Kingdom	Stroke	Cost-utility analysis	Payer	QALY
Shen et al. [52]	2019	China	Stroke	Cost-effectiveness analysis	Healthcare payer	Barthel, Berg and NIHSS ^f^
Tam et al. [53]	2018	Canada	Stroke	Cost-effectiveness analysis	Healthcare payer	Hospitalization day avoided
Tosh et al. [54]	2014	United Kingdom	Multiple sclerosis	Cost-utility analysis	Payer	QALY
Turner-Stokes et al. [16]	2016	United Kingdom	Heterogeneous group	Partial evaluation	Societal	
Wang et al. [55]	2017	Taiwan	Stroke	Partial evaluation	Healthcare payer	-

^a^ QALY: quality-adjusted life-year; ^b^ NEADL: Nottingham Extended Activities for Daily Life; ^c^ HRQL: health-related quality of life; ^d^ CCP: children with cerebral palsy; ^e^ ALS: amyotrophic lateral sclerosis; ^f^ NIHSS: US National Institutes of Health Stroke Scale; ACU: cost-utility analysis; ACE: cost-effectiveness analysis; ACB: cost-benefit analysis; AMC: cost minimization analysis.

## Data Availability

The data presented in this study are available on request from the corresponding author.
